# Crystal structures of *fac*-tri­chlorido­tris­(tri­methyl­phosphane-κ*P*)rhodium(III) monohydrate and *fac*-tri­chlorido­tris­(tri­methyl­phosphane-κ*P*)rhodium(III) methanol hemisolvate: rhodium structures that are isotypic with their iridium analogs

**DOI:** 10.1107/S2056989015001516

**Published:** 2015-01-31

**Authors:** Joseph S. Merola, Marion A. Franks

**Affiliations:** a Department of Chemistry 0212, Virginia Tech, Blacksburg, VA 24061, USA

**Keywords:** crystal structure, iridium, rhodium, phosphane ligands, isotypism

## Abstract

The crystal structures of two solvates of *fac*-tri­chlorido­tris­(tri­methyl­phosphane-κ*P*)rhodium(III) are reported, *i.e.* one with water in the crystal lattice, *fac*-[RhCl_3_(Me_3_P)_3_]·H_2_O, and one with methanol in the crystal lattice, *fac*-[RhCl_3_(Me_3_P)_3_]·0.5CH_3_OH. These rhodium compounds exhibit distorted octahedral coordination spheres at the metal and are isotypic with the analogous iridium compounds previously reported by us [Merola *et al.* (2013 ▸). *Polyhedron*, **54**, 67–73]. Comparison is made between the rhodium and iridium compounds, highlighting their isostructural relationships.

## Abstract 2

The structures of two solvates (water and methanol) of the facial isomer of (Me_3_P)_3_RhCl_3_ are reported and compared with previously published facial (Me_3_P)_3_IrCl_3_ solvates with which they are isostructural and isomorphous.

## Chemical context   

Phosphane complexes of noble metals, especially those of rhodium and iridium, have proven to be important in catalysis as well as in studying fundamental reactions at metal surfaces. Chlorido compounds of rhodium and iridium with phosphane ligands provide important starting materials for other metal complexes of that family through replacement of the chlorine. For example, we have shown that (Me_3_P)_3_IrCl_3_ can be converted into (Me_3_P)_3_IrMe_3_ through reaction with methyl­magnesiumchloride. This tri­methyl­iridium compound can, in turn, be used to study organometallic reactions at the irid­ium(III) atom (Merola *et al.*, 2013 ▸). Thus, the fundamental study of crystal structures of phosphane–chlorido complexes of iridium and rhodium is important to help understand the structures, the bonding and the stereochemistry of this class of compounds. This paper adds to the body of knowledge of rhodium complexes that complement the already published structures of the analogous iridium compounds. It contributes to the information on crystal structures of *L*_3_ *M*Cl_3_ compounds, comparing the rhodium structures to the iridium structures as well as confirming the nature of solvate formation in both the iridium and rhodium structures.
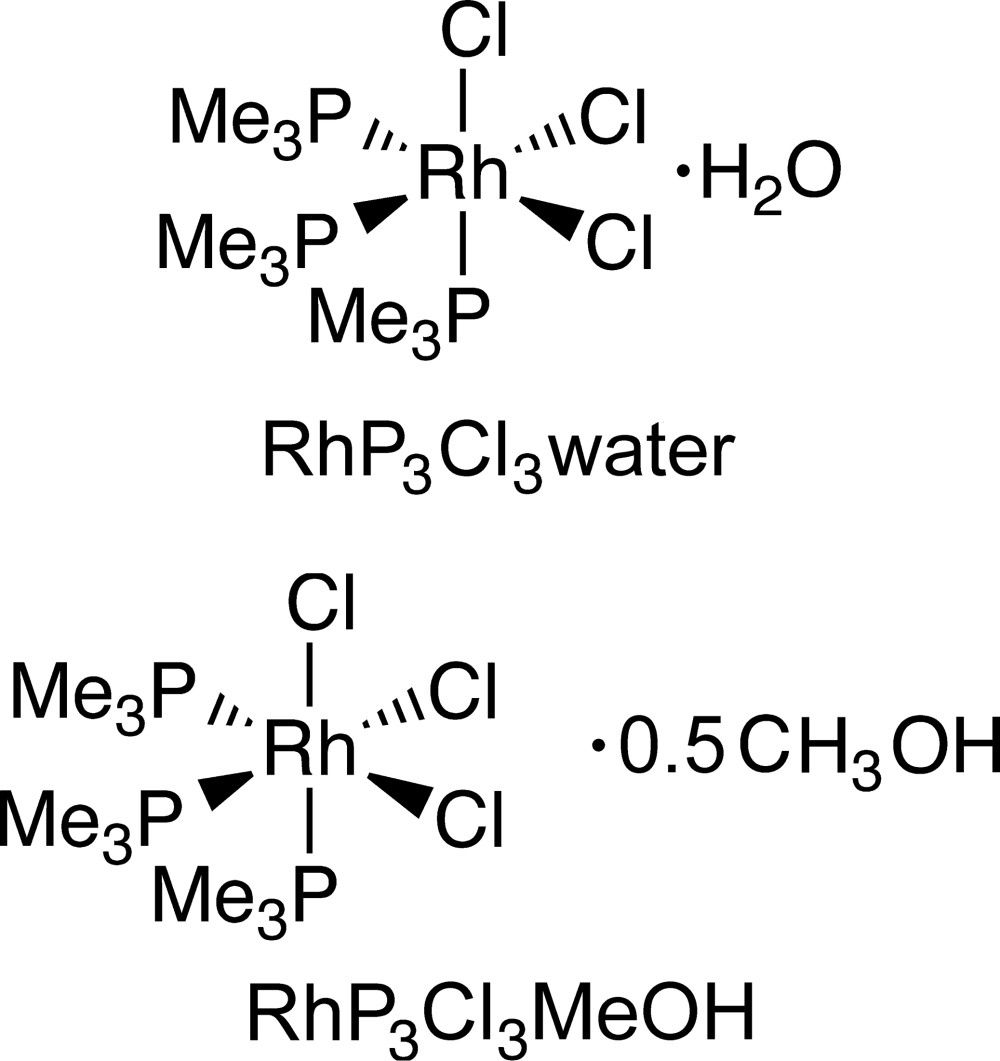


## Structural commentary   

The title complexes *fac*-tri­chlorido­tris­(tri­methyl­phosphane-κ*P*)rhodium(III) monohydrate, **RhP_3_Cl_3_water**, and *fac*-tri­chlorido­tris­(tri­methyl­phosphane-κ*P*)rhodium(III) methanol hemihydrate, **RhP_3_Cl_3_MeOH**, are isotypic with their iridium counterparts (CCDC 896072, 896073; Merola *et al.*, 2013 ▸). Isotypism in rhodium and iridium complexes is not unusual, largely owing to the lanthanide contraction resulting in very similar radii for both second- and third-row transition elements (Cordero *et al.*, 2008 ▸).

**Figure 1:**
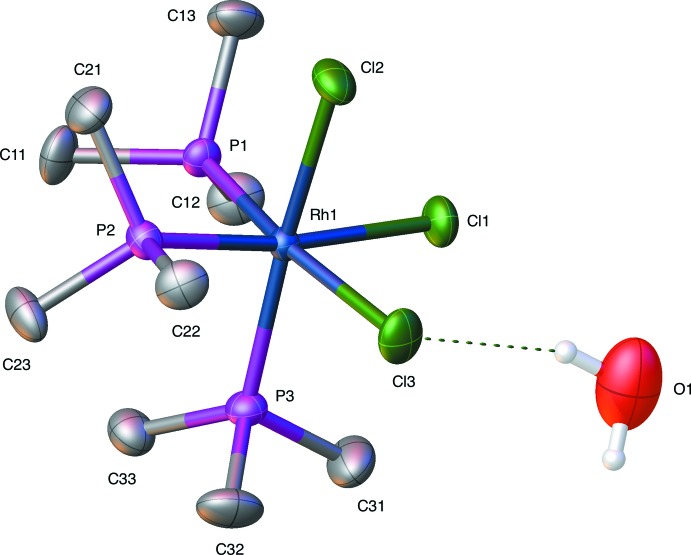
Displacement ellipsoid (50% probability level) rendering of the *fac*-tri­chlorido­tris­(tri­methyl­phosphane)rhodium–water compound, **RhP_3_Cl_3_water**.

**Figure 2:**
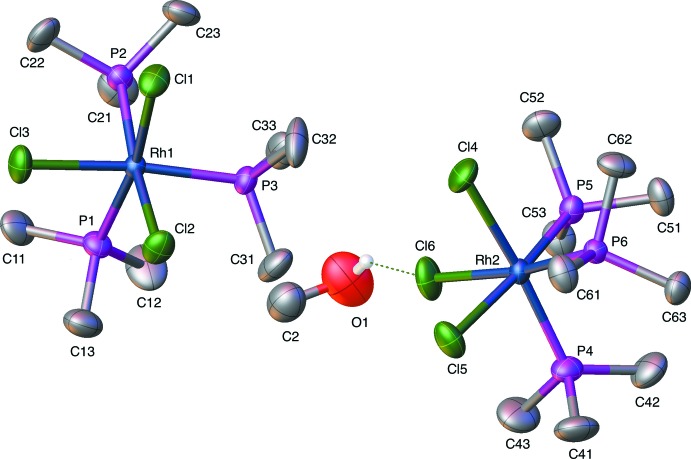
Displacement ellipsoid (50% probability level) rendering of the *fac*-tri­chlorido­tris­(tri­methyl­phosphane)rhodium–0.5(methanol) compound, **RhP_3_Cl_3_MeOH.**

Fig. 1 ▸ is a displacement ellipsoid rendering of compound **RhP_3_Cl_3_water** and Fig. 2 ▸ is a displacement ellipsoid rendering of compound **RhP_3_Cl_3_MeOH**. For compounds **RhP_3_Cl_3_water** and **RhP_3_Cl_3_MeOH** reported here, the comparison with their iridium analogs can be found in Tables 1 ▸ and 2 ▸ which list the corresponding unit-cell parameters for the rhodium and iridium water solvates (Table 1 ▸) and the rhodium and iridium methanol solvate (Table 2 ▸). The iridium compounds show a very slight lengthening of the unit-cell dimensions compared to rhodium but they are clearly isotypic overall. Table 3 ▸ lists the important bond lengths for **RhP_3_Cl_3_water** and **IrP_3_Cl_3_water** while Table 4 ▸ lists these for **RhP_3_Cl_3_MeOH** and **IrP_3_Cl_3_MeOH**. Bond-length comparisons show little significant difference between the rhodium and iridium analogs.

## Supra­molecular features   

**Figure 3:**
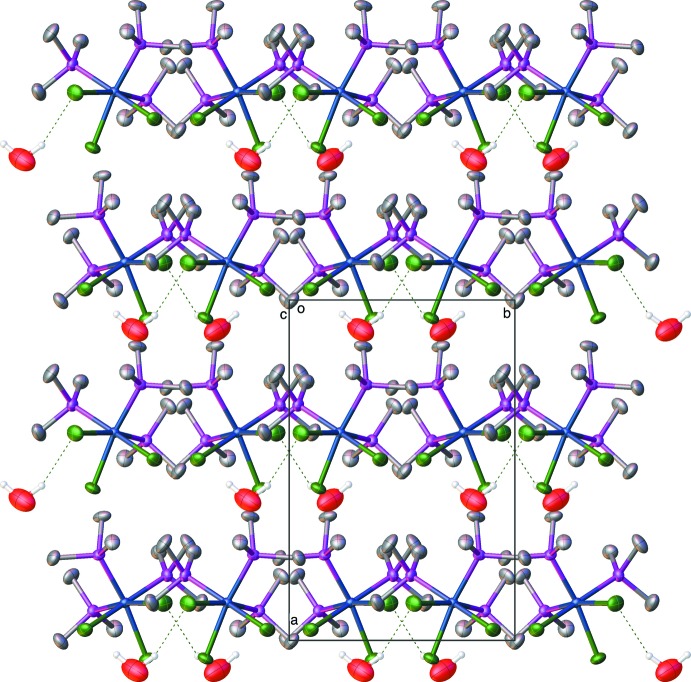
Packing diagram of the *fac*-tri­chlorido­tris­(tri­methyl­phosphane)rhodium–water compound, **RhP_3_Cl_3_water**, viewed down the *c* axis, showing the alternating layers of complex and water mol­ecules. Hydrogen atoms except for water H atoms are omitted for clarity.

**Figure 4:**
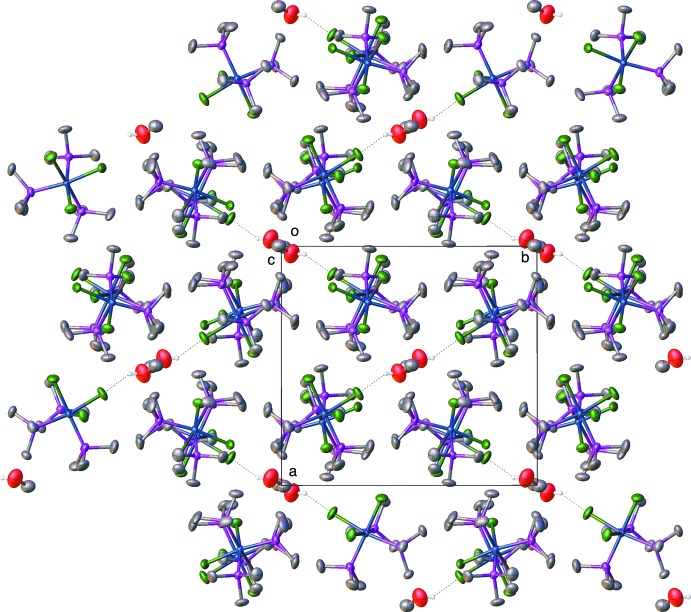
Packing diagram of the *fac*-tri­chlorido­tris­(tri­methyl­phosphane)rhodium–0.5(methanol) compound, **RhP_3_Cl_3_MeOH**, viewed down the *c* axis, showing the methanol-containing channel in the structure. H atoms, except for water H atoms, a omitted for clarity.

It is not surprising that *fac*-tris­(tri­methyl­phosphane)tri­chloroidium(III) and -rhodium(III) complexes form lattice solvates since the shape of the individual mol­ecules leads to packing with voids in the lattice. Thus, every structure we have determined with the iridium compounds, as well as the ones reported here, contains a solvent. In the case of the water solvate, Fig. 3 ▸ shows the packing diagram for **RhP_3_Cl_3_water** looking down the *c* axis. One can see that the packing involves alternating layers of rhodium mol­ecules and water mol­ecules. The water mol­ecules show close, hydrogen-bonding inter­actions (Table 5 ▸) between the water and the chlorines on one layer of the rhodium compound as well as close C—H⋯O inter­actions between the phosphane methyl groups and the water oxygen. One should not make much of the hydrogen positions on the water since, although they were originally found in difference maps, the O—H bond lengths and the H—O—H angle were restrained with DFIX and DANG commands (Sheldrick, 2015 ▸). Fig. 4 ▸ shows the packing diagram for **RhP_3_Cl_3_MeOH**, looking down the *c* axis, illustrating the O—H⋯Cl hydrogen bonding (Table 6 ▸) and the location of the methanol mol­ecules in a channel in the crystal.

## Database survey   

A search of the Cambridge Structural Database (Groom & Allen, 2014 ▸) surprisingly shows very few structurally characterized tri­chlorido­tris­phosphaneiridium or rhodium compounds. In the case of iridium, beside the structures we recently published (CCDC 896072–896076; Merola *et al.*, 2013 ▸), there are only three other P_3_IrCl_3_ compounds in the database – the *mer* and *fac* isomers with P = phenyldi­methyl­phosphane (refcodes CTPIRA01, CTPIRC: Marsh, 1997 ▸; Robertson & Tucker, 1981 ▸) and one entry where P_3_ is *cis*,*cis*-1,3,5-tris­(di­phenyl­phosphino)cyclo­hexane (refcode LEXFAV; Mayer *et al.*, 1994 ▸). For rhodium, P_3_RhCl_3_ structur­ally characterized compounds are also rare with one mixed-ligand complex (two tri-*n*-butyl­phosphane ligands and one tri­methyl­phosphite ligand; refcode CBPMRH; Allen *et al.*, 1970 ▸), a complex with 3 hy­droxy­methyl­phosphane ligands (CCDC 189926; Raghuraman *et al.*, 2002 ▸), a complex with the tripodal ligand, 1,1,1-tris­(di­methyl­phosphinometh­yl)ethane (refcode NAHXID; Suzuki *et al.*, 1996 ▸), a complex with the tridentate ligand, 1,5,9-tris­(2-prop­yl)-1,5,9-triphospha­cyclo­dodecane (refcode NOLPIN; Edwards *et al.*, 1997 ▸), a *mer*-tris-di­methyl­phenyl­phosphane compound (CCDC 247871; Parsons *et al.*, 2004 ▸) and a *mer*-tris-di­ethyl­phenyl­phosphane compound (refcode TCPERH; Skapski & Stephens, 1973 ▸). Of those, the only directly comparable structures are the *mer* isomer complexes of rhodium and iridium with di­methyl­phenyl­phosphane ligands and those two are indeed isostructural with each other.

## Synthesis and crystallization   

The rhodium complexes described herein could not be characterized spectroscopically as pure materials, but were isolated as crystals from complex mixtures. In contrast to the iridium complex [IrCOD(PMe_3_)_3_]Cl (COD = cyclo­octa­diene) (Frazier & Merola, 1992 ▸) which is the starting material for much of our iridium work, attempts to synthesize the analogous rhodium compound met with no success. Reaction between various Rh^I^ olefin complexes, including COD, especially in di­chloro­methane solvent, led to complex mixtures of Rh(PMe_3_)_*n*_ compounds in all cases. That these compounds are compounds of Rh is clearly seen in the Rh–P chemical coupling in the complicated ^31^P NMR spectra. Attempts at extracting a pure compound from the complex mixture with various solvents including di­chloro­methane, water, methanol and acetone did not yield clean materials. Following extraction, the solutions were allowed to sit in the open air for several days and, in the case of water and methanol, a few crystals suitable for X-ray crystallography were formed and used for the data collection described in this communication.

## Refinement   

Crystal data, data collection and structure refinement details are summarized in Table 7 ▸. The hydrogens on the lattice water mol­ecule in **RhP_3_Cl_3_water** were initially assigned based on residual electron density but were then restrained with DFIX and DANG instructions in *SHELXL* (Sheldrick, 2015 ▸) during refinement.

## Supplementary Material

Crystal structure: contains datablock(s) RhP3Cl3water, RhP3Cl3MeOH. DOI: 10.1107/S2056989015001516/pk2543sup1.cif

Supporting information file. DOI: 10.1107/S2056989015001516/pk2543RhP3Cl3watersup4.mol

Structure factors: contains datablock(s) RhP3Cl3MeOH. DOI: 10.1107/S2056989015001516/pk2543RhP3Cl3MeOHsup3.hkl

Supporting information file. DOI: 10.1107/S2056989015001516/pk2543RhP3Cl3MeOHsup5.mol

CCDC references: 1045021, 1045022

Additional supporting information:  crystallographic information; 3D view; checkCIF report

## Figures and Tables

**Table 1:** Comparison of unit-cell dimensions (, ) for water solvate complexes **RhP_3_Cl_3_water** and **IrP_3_Cl_3_water**

Compound	space group	*a*	*b*	*c*	
**RhP_3_Cl_3_water**	*Cc*	15.8650(12)	9.0396(3)	14.8223(18)	120.820(7)
**IrP_3_Cl_3_water**	*Cc*	15.8830(10)	9.0590(10)	14.829(2)	120.530(8)

**Table 2:** Comparison of unit-cell dimensions (, ) for methanol solvate complexes **RhP_3_Cl_3_MeOH** and **IrP_3_Cl_3_MeOH**

Compound	space group	*a*	*b*	*c*	
**RhP_3_Cl_3_MeOH**	*P*2_1_/*n*	16.0993(16)	15.5910(9)	16.4152(14)	115.084(13)
**IrP_3_Cl_3_MeOH**	*P*2_1_/*n*	16.144(3)	15.631(4)	16.469(4)	115.400(17)

**Table 3:** Comparison of significant bond lengths () for **RhP_3_Cl_3_water** and **IrP_3_Cl_3_water**

Compound	*M*P1	*M*P2	*M*P3	*M*Cl1	*M*Cl2	*M*Cl3
**RhP_3_Cl_3_water**	2.279(2)	2.295(3)	2.292(2)	2.450(2)	2.444(3)	2.436(3)
**IrP_3_Cl_3_water**	2.2787(18)	2.2880(19)	2.2912(17)	2.4320(19)	2.4469(18)	2.4451(19)

**Table 4:** Comparison of significant bond lengths () for **RhP_3_Cl_3_MeOH** and **IrP_3_Cl_3_MeOH**

Compound	*M*P1	*M*P2	*M*P3	*M*Cl1	*M*Cl2	*M*Cl3
**RhP_3_Cl_3_MeOH**	2.2824(12)	2.2950(13)	2.2995(12)	2.4246(11)	2.4453(12)	2.4364(12)
	2.2860(13)	2.2954(12)	2.2923(11)	2.4372(12)	2.4476(12)	2.4426(12)
**IrP_3_Cl_3_MeOH**	2.2809(16)	2.2847(17)	2.2964(15)	2.4245(16)	2.4368(17)	2.4394(15)
	2.2932(16)	2.2795(17)	2.2869(16)	2.4442(16)	2.4316(17)	2.4405(17)

**Table 5:** Hydrogen-bond geometry (, ) for **RhP_3_Cl_3_water** [Chem scheme1]

*D*H*A*	*D*H	H*A*	*D* *A*	*D*H*A*
O1H1*B*Cl3	0.97	2.57	3.481	157

**Table 6:** Hydrogen-bond geometry (, ) for **RhP_3_Cl_3_MeOH** [Chem scheme1]

*D*H*A*	*D*H	H*A*	*D* *A*	*D*H*A*
O1H1Cl6^i^	0.82	2.47	3.184(5)	147

Symmetry code: (i) 
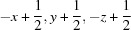
.

**Table 7:** Experimental details

	**RhP_3_Cl_3_water**	**RhP_3_Cl_3_MeOH**
Crystal data		
Chemical formula	[RhCl_3_(C_3_H_9_P)_3_]H_2_O	[RhCl_3_(C_3_H_9_P)_3_]0.5CH_4_O
*M*_r_	455.49	453.50
Crystal system, space group	Monoclinic, *C* *c*	Monoclinic, *P*2_1_/*n*
Temperature (K)	298	298
*a*, *b*, *c* ()	15.8650(12), 9.0396(3), 14.8223(18)	16.0993(16), 15.5910(9), 16.4152(14)
()	120.820(7)	115.084(13)
*V* (^3^)	1825.5(3)	3731.7(5)
*Z*	4	8
Radiation type	Mo *K*	Mo *K*
(mm^1^)	1.62	1.59
Crystal size (mm)	0.4 0.4 0.3	0.6 0.6 0.3

Data collection		
Diffractometer	Siemens P4	Siemens P4
Absorption correction	scan (North *et al.*, 1968 ▸)	scan (North *et al.*, 1968 ▸)
*T*_min_, *T*_max_	0.762, 0.974	0.807, 0.915
No. of measured, independent and observed [*I* > 2(*I*)] reflections	2034, 1784, 1763	5957, 4858, 4171
*R*_int_	0.021	0.034
_max_ ()	25.0	22.5
(sin /)_max_ (^1^)	0.595	0.538

Refinement		
*R*[*F* ^2^ > 2(*F* ^2^)], *wR*(*F* ^2^), *S*	0.023, 0.059, 1.08	0.029, 0.071, 1.08
No. of reflections	1784	4858
No. of parameters	170	328
No. of restraints	5	0
H-atom treatment	H atoms treated by a mixture of independent and constrained refinement	H-atom parameters constrained
_max_, _min_ (e ^3^)	0.47, 0.60	1.03, 0.41
Absolute structure	Classical Flack (1983 ▸) method preferred over Parsons because s.u. lower	
Absolute structure parameter	0.06(3)	

Computer programs: *XSCANS* (Siemens, 1996 ▸), *SHELXS87* and *SHELXS97* (Sheldrick, 2008 ▸), *SHELXL2014* (Sheldrick, 2015 ▸) and *OLEX2* (Dolomanov *et al.*, 2009 ▸).

## supplementary crystallographic information

### Crystal data

**Table:**

[RhCl_3_(C_3_H_9_P)_3_]·0.5CH_4_O	*F*(000) = 1848
*M**_r_* = 453.50	*D*_x_ = 1.614 Mg m^−^^3^
Monoclinic, *P*2_1_/*n*	Mo *K*α radiation, λ = 0.71073 Å
*a* = 16.0993 (16) Å	Cell parameters from 50 reflections
*b* = 15.5910 (9) Å	θ = 3–20°
*c* = 16.4152 (14) Å	µ = 1.59 mm^−^^1^
β = 115.084 (13)°	*T* = 298 K
*V* = 3731.7 (5) Å^3^	Prism, clear light yellow
*Z* = 8	0.6 × 0.6 × 0.3 mm

### Data collection

**Table:**

Siemens P4 diffractometer	4171 reflections with *I* > 2σ(*I*)
Radiation source: fine-focus sealed tube	*R*_int_ = 0.034
Graphite monochromator	θ_max_ = 22.5°, θ_min_ = 1.9°
sea;ed X–ray tube scans	*h* = −1→17
Absorption correction: ψ scan (North *et al.*, 1968)	*k* = −1→16
*T*_min_ = 0.807, *T*_max_ = 0.915	*l* = −17→16
5957 measured reflections	3 standard reflections every 200 reflections
4858 independent reflections	intensity decay: 0.0(2)

### Refinement

**Table:**

Refinement on *F*^2^	Secondary atom site location: difference Fourier map
Least-squares matrix: full	Hydrogen site location: inferred from neighbouring sites
*R*[*F*^2^ > 2σ(*F*^2^)] = 0.029	H-atom parameters constrained
*wR*(*F*^2^) = 0.071	*w* = 1/[σ^2^(*F*_o_^2^) + (0.0286*P*)^2^ + 4.1793*P*] where *P* = (*F*_o_^2^ + 2*F*_c_^2^)/3
*S* = 1.08	(Δ/σ)_max_ = 0.003
4858 reflections	Δρ_max_ = 1.03 e Å^−^^3^
328 parameters	Δρ_min_ = −0.41 e Å^−^^3^
0 restraints	Extinction correction: *SHELXL2014* (Sheldrick, 2015), Fc^*^=kFc[1+0.001xFc^2^λ^3^/sin(2θ)]^-1/4^
Primary atom site location: structure-invariant direct methods	Extinction coefficient: 0.00519 (17)

### Special details

**Table:**

Geometry. All e.s.d.'s (except the e.s.d. in the dihedral angle between two l.s. planes) are estimated using the full covariance matrix. The cell e.s.d.'s are taken into account individually in the estimation of e.s.d.'s in distances, angles and torsion angles; correlations between e.s.d.'s in cell parameters are only used when they are defined by crystal symmetry. An approximate (isotropic) treatment of cell e.s.d.'s is used for estimating e.s.d.'s involving l.s. planes.
Refinement. Refinement of *F*^2^ against ALL reflections. The weighted *R*-factor *wR* and goodness of fit *S* are based on *F*^2^, conventional *R*-factors *R* are based on *F*, with *F* set to zero for negative *F*^2^. The threshold expression of *F*^2^ > σ(*F*^2^) is used only for calculating *R*-factors(gt) *etc*. and is not relevant to the choice of reflections for refinement. *R*-factors based on *F*^2^ are statistically about twice as large as those based on *F*, and *R*- factors based on ALL data will be even larger.

### Fractional atomic coordinates and isotropic or equivalent isotropic displacement parameters (Å^2^)

**Table:**

	*x*	*y*	*z*	*U*_iso_*/*U*_eq_	
Rh1	0.76635 (2)	0.66132 (2)	0.08786 (2)	0.02237 (12)	
Cl1	0.82866 (8)	0.79954 (7)	0.07477 (9)	0.0435 (3)	
Cl2	0.65651 (8)	0.67764 (8)	−0.06932 (7)	0.0445 (3)	
Cl3	0.87487 (8)	0.60045 (8)	0.03606 (8)	0.0449 (3)	
P1	0.72796 (9)	0.52126 (7)	0.09374 (8)	0.0361 (3)	
P2	0.87465 (8)	0.66934 (7)	0.23406 (8)	0.0308 (3)	
P3	0.64873 (8)	0.71971 (7)	0.11404 (8)	0.0314 (3)	
C11	0.8230 (4)	0.4462 (3)	0.1382 (4)	0.0583 (15)	
H11A	0.8605	0.4524	0.1061	0.087*	
H11B	0.7996	0.3887	0.1311	0.087*	
H11C	0.8590	0.4577	0.2009	0.087*	
C12	0.6631 (5)	0.4911 (4)	0.1567 (5)	0.076 (2)	
H12A	0.6949	0.5102	0.2178	0.114*	
H12B	0.6565	0.4298	0.1557	0.114*	
H12C	0.6035	0.5172	0.1299	0.114*	
C13	0.6596 (4)	0.4779 (3)	−0.0171 (4)	0.0614 (16)	
H13A	0.6011	0.5060	−0.0426	0.092*	
H13B	0.6509	0.4175	−0.0125	0.092*	
H13C	0.6907	0.4872	−0.0550	0.092*	
C21	0.8719 (4)	0.5969 (4)	0.3203 (3)	0.0588 (15)	
H21A	0.8132	0.6008	0.3222	0.088*	
H21B	0.9191	0.6127	0.3778	0.088*	
H21C	0.8818	0.5391	0.3063	0.088*	
C22	0.9893 (3)	0.6550 (4)	0.2430 (4)	0.0567 (15)	
H22A	0.9979	0.5962	0.2310	0.085*	
H22B	1.0329	0.6699	0.3027	0.085*	
H22C	0.9983	0.6914	0.2002	0.085*	
C23	0.8810 (4)	0.7718 (3)	0.2882 (4)	0.0536 (14)	
H23A	0.8582	0.8160	0.2434	0.080*	
H23B	0.9436	0.7839	0.3282	0.080*	
H23C	0.8445	0.7699	0.3217	0.080*	
C31	0.5349 (3)	0.6773 (4)	0.0457 (4)	0.0572 (15)	
H31A	0.5217	0.6830	−0.0169	0.086*	
H31B	0.4902	0.7086	0.0580	0.086*	
H31C	0.5327	0.6178	0.0597	0.086*	
C32	0.6344 (4)	0.8316 (3)	0.0863 (5)	0.071 (2)	
H32A	0.6892	0.8621	0.1238	0.107*	
H32B	0.5835	0.8537	0.0958	0.107*	
H32C	0.6228	0.8391	0.0243	0.107*	
C33	0.6502 (4)	0.7160 (4)	0.2248 (3)	0.0554 (15)	
H33A	0.6542	0.6574	0.2442	0.083*	
H33B	0.5950	0.7413	0.2227	0.083*	
H33C	0.7023	0.7473	0.2664	0.083*	
Rh2	0.29937 (2)	0.83004 (2)	0.11738 (2)	0.02531 (12)	
Cl4	0.44698 (8)	0.89262 (9)	0.14246 (9)	0.0497 (3)	
Cl5	0.29238 (9)	0.77329 (8)	−0.02441 (8)	0.0480 (3)	
Cl6	0.38403 (10)	0.69898 (8)	0.18403 (9)	0.0534 (4)	
P4	0.15796 (9)	0.76893 (8)	0.07421 (9)	0.0412 (3)	
P5	0.31781 (8)	0.86191 (7)	0.26057 (7)	0.0309 (3)	
P6	0.24255 (8)	0.96155 (7)	0.05702 (7)	0.0277 (3)	
C41	0.0844 (4)	0.7767 (4)	−0.0452 (4)	0.0686 (17)	
H41A	0.0786	0.8357	−0.0634	0.103*	
H41B	0.0250	0.7539	−0.0571	0.103*	
H41C	0.1108	0.7445	−0.0782	0.103*	
C42	0.0818 (4)	0.8051 (4)	0.1233 (4)	0.0610 (16)	
H42A	0.1085	0.7920	0.1864	0.092*	
H42B	0.0237	0.7766	0.0943	0.092*	
H42C	0.0729	0.8660	0.1152	0.092*	
C43	0.1622 (5)	0.6541 (3)	0.0936 (5)	0.0735 (19)	
H43A	0.1949	0.6269	0.0635	0.110*	
H43B	0.1010	0.6317	0.0706	0.110*	
H43C	0.1931	0.6429	0.1570	0.110*	
C51	0.2460 (4)	0.9416 (3)	0.2801 (3)	0.0477 (13)	
H51A	0.2562	0.9965	0.2596	0.072*	
H51B	0.2612	0.9444	0.3432	0.072*	
H51C	0.1828	0.9258	0.2477	0.072*	
C52	0.4313 (3)	0.9011 (4)	0.3307 (3)	0.0522 (14)	
H52A	0.4761	0.8607	0.3302	0.078*	
H52B	0.4376	0.9082	0.3911	0.078*	
H52C	0.4406	0.9553	0.3080	0.078*	
C53	0.3059 (4)	0.7705 (3)	0.3231 (3)	0.0480 (13)	
H53A	0.2448	0.7482	0.2937	0.072*	
H53B	0.3177	0.7882	0.3830	0.072*	
H53C	0.3491	0.7269	0.3256	0.072*	
C61	0.2455 (4)	0.9750 (3)	−0.0508 (3)	0.0470 (13)	
H61A	0.3062	0.9631	−0.0452	0.071*	
H61B	0.2292	1.0329	−0.0711	0.071*	
H61C	0.2028	0.9362	−0.0934	0.071*	
C62	0.3089 (4)	1.0522 (3)	0.1198 (3)	0.0516 (14)	
H62A	0.3124	1.0511	0.1797	0.077*	
H62B	0.2798	1.1045	0.0905	0.077*	
H62C	0.3696	1.0493	0.1226	0.077*	
C63	0.1272 (3)	0.9971 (3)	0.0347 (3)	0.0425 (12)	
H63A	0.0832	0.9593	−0.0082	0.064*	
H63B	0.1180	1.0543	0.0108	0.064*	
H63C	0.1196	0.9965	0.0896	0.064*	
O1	0.0306 (5)	1.0413 (3)	0.1833 (4)	0.1076 (18)	
H1	0.0327	1.0913	0.1996	0.161*	
C2	−0.0030 (4)	0.9903 (4)	0.2311 (4)	0.0705 (17)	
H2A	−0.0355	0.9425	0.1946	0.106*	
H2B	−0.0439	1.0232	0.2476	0.106*	
H2C	0.0471	0.9696	0.2845	0.106*	

### Atomic displacement parameters (Å^2^)

**Table:**

	*U*^11^	*U*^22^	*U*^33^	*U*^12^	*U*^13^	*U*^23^
Rh1	0.0230 (2)	0.02200 (19)	0.02360 (19)	0.00153 (14)	0.01137 (15)	0.00271 (14)
Cl1	0.0405 (7)	0.0301 (6)	0.0631 (8)	−0.0004 (5)	0.0251 (6)	0.0148 (6)
Cl2	0.0390 (7)	0.0641 (8)	0.0261 (6)	0.0069 (6)	0.0096 (5)	0.0060 (5)
Cl3	0.0452 (7)	0.0563 (8)	0.0423 (7)	0.0173 (6)	0.0273 (6)	0.0041 (6)
P1	0.0411 (7)	0.0244 (6)	0.0434 (7)	−0.0039 (5)	0.0186 (6)	−0.0009 (5)
P2	0.0303 (6)	0.0311 (6)	0.0282 (6)	0.0023 (5)	0.0095 (5)	0.0005 (5)
P3	0.0265 (6)	0.0341 (6)	0.0373 (7)	0.0029 (5)	0.0172 (5)	0.0018 (5)
C11	0.065 (4)	0.027 (3)	0.068 (4)	0.011 (3)	0.015 (3)	0.004 (3)
C12	0.102 (5)	0.042 (3)	0.114 (6)	−0.013 (3)	0.076 (5)	0.013 (3)
C13	0.062 (4)	0.043 (3)	0.064 (4)	−0.009 (3)	0.012 (3)	−0.015 (3)
C21	0.078 (4)	0.060 (4)	0.036 (3)	0.002 (3)	0.022 (3)	0.009 (3)
C22	0.025 (3)	0.081 (4)	0.050 (3)	0.010 (3)	0.002 (2)	0.003 (3)
C23	0.057 (3)	0.044 (3)	0.046 (3)	−0.009 (3)	0.008 (3)	−0.018 (3)
C31	0.029 (3)	0.086 (4)	0.054 (3)	−0.004 (3)	0.016 (3)	−0.004 (3)
C32	0.069 (4)	0.042 (3)	0.131 (6)	0.026 (3)	0.070 (4)	0.022 (3)
C33	0.049 (3)	0.081 (4)	0.043 (3)	0.007 (3)	0.027 (3)	−0.010 (3)
Rh2	0.0255 (2)	0.0244 (2)	0.0276 (2)	0.00468 (14)	0.01264 (16)	0.00091 (14)
Cl4	0.0295 (6)	0.0641 (8)	0.0616 (8)	0.0000 (6)	0.0253 (6)	0.0014 (7)
Cl5	0.0720 (9)	0.0401 (7)	0.0366 (7)	0.0102 (6)	0.0275 (6)	−0.0030 (5)
Cl6	0.0707 (9)	0.0416 (7)	0.0518 (8)	0.0308 (7)	0.0297 (7)	0.0140 (6)
P4	0.0372 (7)	0.0310 (7)	0.0511 (8)	−0.0076 (6)	0.0145 (6)	−0.0006 (6)
P5	0.0313 (7)	0.0342 (7)	0.0281 (6)	0.0046 (5)	0.0134 (5)	0.0024 (5)
P6	0.0283 (6)	0.0244 (6)	0.0297 (6)	0.0011 (5)	0.0118 (5)	0.0003 (5)
C41	0.050 (3)	0.068 (4)	0.062 (4)	−0.019 (3)	−0.001 (3)	−0.013 (3)
C42	0.040 (3)	0.071 (4)	0.079 (4)	−0.009 (3)	0.031 (3)	0.004 (3)
C43	0.079 (5)	0.033 (3)	0.101 (5)	−0.013 (3)	0.032 (4)	0.002 (3)
C51	0.061 (3)	0.047 (3)	0.043 (3)	0.016 (3)	0.030 (3)	0.000 (2)
C52	0.045 (3)	0.066 (4)	0.033 (3)	−0.003 (3)	0.004 (2)	0.002 (3)
C53	0.063 (3)	0.044 (3)	0.045 (3)	0.005 (3)	0.031 (3)	0.013 (2)
C61	0.064 (3)	0.039 (3)	0.047 (3)	0.011 (3)	0.032 (3)	0.009 (2)
C62	0.058 (3)	0.030 (3)	0.053 (3)	−0.013 (2)	0.012 (3)	−0.006 (2)
C63	0.035 (3)	0.042 (3)	0.050 (3)	0.011 (2)	0.017 (2)	0.006 (2)
O1	0.145 (5)	0.095 (4)	0.077 (3)	−0.022 (4)	0.040 (3)	−0.020 (3)
C2	0.063 (4)	0.084 (5)	0.063 (4)	−0.003 (4)	0.026 (3)	0.001 (4)

### Geometric parameters (Å, º)

**Table:**

Rh1—Cl1	2.4248 (11)	Rh2—P4	2.2857 (13)
Rh1—Cl2	2.4455 (12)	Rh2—P5	2.2952 (12)
Rh1—Cl3	2.4363 (12)	Rh2—P6	2.2922 (11)
Rh1—P1	2.2825 (12)	P4—C41	1.814 (6)
Rh1—P2	2.2951 (12)	P4—C42	1.819 (5)
Rh1—P3	2.2998 (12)	P4—C43	1.815 (5)
P1—C11	1.816 (5)	P5—C51	1.815 (5)
P1—C12	1.816 (5)	P5—C52	1.804 (5)
P1—C13	1.811 (5)	P5—C53	1.812 (5)
P2—C21	1.827 (5)	P6—C61	1.802 (5)
P2—C22	1.803 (5)	P6—C62	1.808 (5)
P2—C23	1.810 (5)	P6—C63	1.820 (4)
P3—C31	1.820 (5)	C41—H41A	0.9600
P3—C32	1.793 (5)	C41—H41B	0.9600
P3—C33	1.810 (5)	C41—H41C	0.9600
C11—H11A	0.9600	C42—H42A	0.9600
C11—H11B	0.9600	C42—H42B	0.9600
C11—H11C	0.9600	C42—H42C	0.9600
C12—H12A	0.9600	C43—H43A	0.9600
C12—H12B	0.9600	C43—H43B	0.9600
C12—H12C	0.9600	C43—H43C	0.9600
C13—H13A	0.9600	C51—H51A	0.9600
C13—H13B	0.9600	C51—H51B	0.9600
C13—H13C	0.9600	C51—H51C	0.9600
C21—H21A	0.9600	C52—H52A	0.9600
C21—H21B	0.9600	C52—H52B	0.9600
C21—H21C	0.9600	C52—H52C	0.9600
C22—H22A	0.9600	C53—H53A	0.9600
C22—H22B	0.9600	C53—H53B	0.9600
C22—H22C	0.9600	C53—H53C	0.9600
C23—H23A	0.9600	C61—H61A	0.9600
C23—H23B	0.9600	C61—H61B	0.9600
C23—H23C	0.9600	C61—H61C	0.9600
C31—H31A	0.9600	C62—H62A	0.9600
C31—H31B	0.9600	C62—H62B	0.9600
C31—H31C	0.9600	C62—H62C	0.9600
C32—H32A	0.9600	C63—H63A	0.9600
C32—H32B	0.9600	C63—H63B	0.9600
C32—H32C	0.9600	C63—H63C	0.9600
C33—H33A	0.9600	O1—H1	0.8200
C33—H33B	0.9600	O1—C2	1.379 (7)
C33—H33C	0.9600	C2—H2A	0.9600
Rh2—Cl4	2.4371 (12)	C2—H2B	0.9600
Rh2—Cl5	2.4477 (12)	C2—H2C	0.9600
Rh2—Cl6	2.4424 (12)		
			
Cl1—Rh1—Cl2	87.44 (4)	P4—Rh2—Cl5	85.19 (5)
Cl1—Rh1—Cl3	86.01 (4)	P4—Rh2—Cl6	94.79 (5)
Cl3—Rh1—Cl2	88.69 (4)	P4—Rh2—P5	95.00 (5)
P1—Rh1—Cl1	169.61 (4)	P4—Rh2—P6	94.38 (4)
P1—Rh1—Cl2	93.24 (5)	P5—Rh2—Cl4	92.68 (5)
P1—Rh1—Cl3	83.64 (5)	P5—Rh2—Cl5	170.38 (4)
P1—Rh1—P2	96.15 (4)	P5—Rh2—Cl6	85.24 (4)
P1—Rh1—P3	96.41 (4)	P6—Rh2—Cl4	84.07 (4)
P2—Rh1—Cl1	83.39 (4)	P6—Rh2—Cl5	93.63 (4)
P2—Rh1—Cl2	170.60 (4)	P6—Rh2—Cl6	170.61 (5)
P2—Rh1—Cl3	92.69 (4)	P6—Rh2—P5	95.95 (4)
P2—Rh1—P3	95.95 (4)	C41—P4—Rh2	114.5 (2)
P3—Rh1—Cl1	93.96 (4)	C41—P4—C42	101.8 (3)
P3—Rh1—Cl2	82.62 (4)	C41—P4—C43	102.4 (3)
P3—Rh1—Cl3	171.30 (4)	C42—P4—Rh2	120.21 (19)
C11—P1—Rh1	115.90 (18)	C43—P4—Rh2	113.5 (2)
C11—P1—C12	101.2 (3)	C43—P4—C42	102.2 (3)
C12—P1—Rh1	120.2 (2)	C51—P5—Rh2	120.87 (17)
C13—P1—Rh1	112.00 (19)	C52—P5—Rh2	112.52 (18)
C13—P1—C11	102.3 (3)	C52—P5—C51	101.8 (3)
C13—P1—C12	103.0 (3)	C52—P5—C53	103.1 (2)
C21—P2—Rh1	121.30 (19)	C53—P5—Rh2	114.25 (18)
C22—P2—Rh1	112.11 (18)	C53—P5—C51	102.1 (2)
C22—P2—C21	102.8 (3)	C61—P6—Rh2	110.90 (16)
C22—P2—C23	103.1 (3)	C61—P6—C62	102.3 (2)
C23—P2—Rh1	114.92 (18)	C61—P6—C63	102.5 (2)
C23—P2—C21	100.4 (3)	C62—P6—Rh2	114.98 (17)
C31—P3—Rh1	115.75 (18)	C62—P6—C63	100.5 (2)
C32—P3—Rh1	111.43 (18)	C63—P6—Rh2	123.06 (16)
C32—P3—C31	102.1 (3)	P4—C41—H41A	109.5
C32—P3—C33	103.4 (3)	P4—C41—H41B	109.5
C33—P3—Rh1	120.93 (18)	P4—C41—H41C	109.5
C33—P3—C31	100.9 (3)	H41A—C41—H41B	109.5
P1—C11—H11A	109.5	H41A—C41—H41C	109.5
P1—C11—H11B	109.5	H41B—C41—H41C	109.5
P1—C11—H11C	109.5	P4—C42—H42A	109.5
H11A—C11—H11B	109.5	P4—C42—H42B	109.5
H11A—C11—H11C	109.5	P4—C42—H42C	109.5
H11B—C11—H11C	109.5	H42A—C42—H42B	109.5
P1—C12—H12A	109.5	H42A—C42—H42C	109.5
P1—C12—H12B	109.5	H42B—C42—H42C	109.5
P1—C12—H12C	109.5	P4—C43—H43A	109.5
H12A—C12—H12B	109.5	P4—C43—H43B	109.5
H12A—C12—H12C	109.5	P4—C43—H43C	109.5
H12B—C12—H12C	109.5	H43A—C43—H43B	109.5
P1—C13—H13A	109.5	H43A—C43—H43C	109.5
P1—C13—H13B	109.5	H43B—C43—H43C	109.5
P1—C13—H13C	109.5	P5—C51—H51A	109.5
H13A—C13—H13B	109.5	P5—C51—H51B	109.5
H13A—C13—H13C	109.5	P5—C51—H51C	109.5
H13B—C13—H13C	109.5	H51A—C51—H51B	109.5
P2—C21—H21A	109.5	H51A—C51—H51C	109.5
P2—C21—H21B	109.5	H51B—C51—H51C	109.5
P2—C21—H21C	109.5	P5—C52—H52A	109.5
H21A—C21—H21B	109.5	P5—C52—H52B	109.5
H21A—C21—H21C	109.5	P5—C52—H52C	109.5
H21B—C21—H21C	109.5	H52A—C52—H52B	109.5
P2—C22—H22A	109.5	H52A—C52—H52C	109.5
P2—C22—H22B	109.5	H52B—C52—H52C	109.5
P2—C22—H22C	109.5	P5—C53—H53A	109.5
H22A—C22—H22B	109.5	P5—C53—H53B	109.5
H22A—C22—H22C	109.5	P5—C53—H53C	109.5
H22B—C22—H22C	109.5	H53A—C53—H53B	109.5
P2—C23—H23A	109.5	H53A—C53—H53C	109.5
P2—C23—H23B	109.5	H53B—C53—H53C	109.5
P2—C23—H23C	109.5	P6—C61—H61A	109.5
H23A—C23—H23B	109.5	P6—C61—H61B	109.5
H23A—C23—H23C	109.5	P6—C61—H61C	109.5
H23B—C23—H23C	109.5	H61A—C61—H61B	109.5
P3—C31—H31A	109.5	H61A—C61—H61C	109.5
P3—C31—H31B	109.5	H61B—C61—H61C	109.5
P3—C31—H31C	109.5	P6—C62—H62A	109.5
H31A—C31—H31B	109.5	P6—C62—H62B	109.5
H31A—C31—H31C	109.5	P6—C62—H62C	109.5
H31B—C31—H31C	109.5	H62A—C62—H62B	109.5
P3—C32—H32A	109.5	H62A—C62—H62C	109.5
P3—C32—H32B	109.5	H62B—C62—H62C	109.5
P3—C32—H32C	109.5	P6—C63—H63A	109.5
H32A—C32—H32B	109.5	P6—C63—H63B	109.5
H32A—C32—H32C	109.5	P6—C63—H63C	109.5
H32B—C32—H32C	109.5	H63A—C63—H63B	109.5
P3—C33—H33A	109.5	H63A—C63—H63C	109.5
P3—C33—H33B	109.5	H63B—C63—H63C	109.5
P3—C33—H33C	109.5	C2—O1—H1	109.5
H33A—C33—H33B	109.5	O1—C2—H2A	109.5
H33A—C33—H33C	109.5	O1—C2—H2B	109.5
H33B—C33—H33C	109.5	O1—C2—H2C	109.5
Cl4—Rh2—Cl5	87.35 (5)	H2A—C2—H2B	109.5
Cl4—Rh2—Cl6	86.57 (5)	H2A—C2—H2C	109.5
Cl6—Rh2—Cl5	85.15 (4)	H2B—C2—H2C	109.5
P4—Rh2—Cl4	172.28 (5)		
			
Cl1—Rh1—P1—C11	36.4 (4)	P3—Rh1—P2—C23	−47.9 (2)
Cl1—Rh1—P1—C12	158.6 (3)	Cl4—Rh2—P5—C51	111.9 (2)
Cl1—Rh1—P1—C13	−80.4 (3)	Cl4—Rh2—P5—C52	−8.4 (2)
Cl1—Rh1—P2—C21	166.5 (2)	Cl4—Rh2—P5—C53	−125.53 (19)
Cl1—Rh1—P2—C22	−71.8 (2)	Cl4—Rh2—P6—C61	72.9 (2)
Cl1—Rh1—P2—C23	45.5 (2)	Cl4—Rh2—P6—C62	−42.6 (2)
Cl1—Rh1—P3—C31	130.6 (2)	Cl4—Rh2—P6—C63	−165.5 (2)
Cl1—Rh1—P3—C32	14.5 (3)	Cl5—Rh2—P4—C41	−38.0 (2)
Cl1—Rh1—P3—C33	−107.1 (2)	Cl5—Rh2—P4—C42	−159.7 (2)
Cl2—Rh1—P1—C11	129.9 (2)	Cl5—Rh2—P4—C43	79.1 (3)
Cl2—Rh1—P1—C12	−108.0 (3)	Cl5—Rh2—P6—C61	−14.1 (2)
Cl2—Rh1—P1—C13	13.1 (2)	Cl5—Rh2—P6—C62	−129.6 (2)
Cl2—Rh1—P3—C31	43.7 (2)	Cl5—Rh2—P6—C63	107.5 (2)
Cl2—Rh1—P3—C32	−72.3 (3)	Cl6—Rh2—P4—C41	−122.7 (2)
Cl2—Rh1—P3—C33	166.0 (2)	Cl6—Rh2—P4—C42	115.6 (2)
Cl3—Rh1—P1—C11	41.5 (2)	Cl6—Rh2—P4—C43	−5.6 (3)
Cl3—Rh1—P1—C12	163.7 (3)	Cl6—Rh2—P5—C51	−161.7 (2)
Cl3—Rh1—P1—C13	−75.3 (2)	Cl6—Rh2—P5—C52	77.9 (2)
Cl3—Rh1—P2—C21	−107.9 (2)	Cl6—Rh2—P5—C53	−39.2 (2)
Cl3—Rh1—P2—C22	13.9 (2)	P4—Rh2—P5—C51	−67.3 (2)
Cl3—Rh1—P2—C23	131.1 (2)	P4—Rh2—P5—C52	172.3 (2)
P1—Rh1—P2—C21	−24.0 (2)	P4—Rh2—P5—C53	55.2 (2)
P1—Rh1—P2—C22	97.7 (2)	P4—Rh2—P6—C61	−99.5 (2)
P1—Rh1—P2—C23	−145.0 (2)	P4—Rh2—P6—C62	145.0 (2)
P1—Rh1—P3—C31	−48.7 (2)	P4—Rh2—P6—C63	22.1 (2)
P1—Rh1—P3—C32	−164.8 (3)	P5—Rh2—P4—C41	151.7 (2)
P1—Rh1—P3—C33	73.5 (2)	P5—Rh2—P4—C42	30.0 (2)
P2—Rh1—P1—C11	−50.5 (2)	P5—Rh2—P4—C43	−91.3 (3)
P2—Rh1—P1—C12	71.7 (3)	P5—Rh2—P6—C61	164.96 (19)
P2—Rh1—P1—C13	−167.3 (2)	P5—Rh2—P6—C62	49.5 (2)
P2—Rh1—P3—C31	−145.7 (2)	P5—Rh2—P6—C63	−73.4 (2)
P2—Rh1—P3—C32	98.3 (3)	P6—Rh2—P4—C41	55.3 (2)
P2—Rh1—P3—C33	−23.4 (2)	P6—Rh2—P4—C42	−66.4 (2)
P3—Rh1—P1—C11	−147.2 (2)	P6—Rh2—P4—C43	172.4 (3)
P3—Rh1—P1—C12	−25.0 (3)	P6—Rh2—P5—C51	27.6 (2)
P3—Rh1—P1—C13	96.0 (2)	P6—Rh2—P5—C52	−92.7 (2)
P3—Rh1—P2—C21	73.1 (2)	P6—Rh2—P5—C53	150.16 (19)
P3—Rh1—P2—C22	−165.1 (2)		

### Hydrogen-bond geometry (Å, º)

**Table:**

*D*—H···*A*	*D*—H	H···*A*	*D*···*A*	*D*—H···*A*
O1—H1···Cl6^i^	0.82	2.47	3.184 (5)	147

Symmetry code: (i) −*x*+1/2, *y*+1/2, −*z*+1/2.
